# Implementing practice facilitation in research: how facilitators spend their time guiding practices to improve blood pressure control

**DOI:** 10.1186/s43058-023-00470-y

**Published:** 2023-07-31

**Authors:** Kent F. Sutton, Erica L. Richman, Jennifer R. Rees, Liza L. Pugh-Nicholson, Macie M. Craft, Shannon H. Peaden, Orysya Soroka, Monique Mackey, Doyle M. Cummings, Andrea L. Cherrington, Monika M. Safford, Jacqueline R. Halladay, Southeastern Collaboration to Improve Blood Pressure Writing Group

**Affiliations:** 1 University of North Carolina at Chapel Hill, Cecil G. Sheps Center for Health Services Research, Chapel Hill, NC USA; 2 Duke University School of Medicine, Durham, NC USA; 3 University of North Carolina at Chapel Hill, North Carolina Translational and Clinical Sciences, Chapel Hill, NC USA; 4 University of Alabama at Birmingham, Birmingham, AL USA; 5 Samford University, Birmingham, AL USA; 6 East Carolina University, Greenville, NC USA; 7 Weill Cornell Medicine, New York, NY USA; 8 Area L Area Health Education Center, Rocky Mount, NC USA; 9 Department of Family Medicine, University of North Carolina at Chapel Hill, Chapel Hill, NC USA

**Keywords:** Practice facilitation, Primary care, Quality improvement, Hypertension, Rural health

## Abstract

**Background:**

Practice facilitators (PFs) coach practices through quality improvement (QI) initiatives aimed at enhancing patient outcomes and operational efficiencies. Practice facilitation is a dynamic intervention that, by design, is tailored to practices’ unique needs and contexts. Little research has explored the amount of time PFs spend with practices on QI activities. This short report expands on previously published work that detailed a 12-month practice facilitation intervention as part of the Southeastern Collaboration to Improve Blood Pressure Control (SEC) trial, which focused on improving hypertension control among people living in rural settings in the southeastern USA. This report analyzes data on the time PFs spent to guide 32 primary care practices in implementing QI activities to support enhanced outcomes in patients with high blood pressure.

**Methods:**

The SEC trial employed four certified PFs across all practice sites, who documented time spent: (1) driving to support practices; (2) working on-site with staff and clinicians; and (3) communicating remotely (phone, email, or video conference) with practice members. We analyzed the data using descriptive statistics to help understand time devoted to individual and aggregated tasks. Additionally, we explored correlations between practice characteristics and time spent with PFs.

**Results:**

In aggregate, the PFs completed 416 visits to practices and spent an average of 130 (SD 65) min per visit driving to and from practices. The average time spent on-site per visit with practices was 87 (SD 37) min, while an average of 17 (SD 12) min was spent on individual remote communications. During the 12-month intervention, 1131 remote communications were conducted with practices. PFs spent most of their time with clinical staff members (*n* = 886 instances) or with practice managers alone (*n* = 670 instances) while relatively few on-site visits were conducted with primary care providers alone (*n* = 15). In 19 practices, no communications were solely with providers. No significant correlations were found between time spent on PF activities and a practices’ percent of Medicaid and uninsured patients, staff-provider ratio, or federally qualified health center (FQHC) status.

**Conclusions:**

PFs working with practices serving rural patients with hypertension devote substantial time to driving, highlighting the importance of optimizing a balance between time spent on-site vs. communicating remotely. Most time spent was with clinical staff, not primary care providers. These findings may be useful to researchers and business leaders who design, test, and implement efficient facilitation services.

**Trial registration:**

NIH ClinicalTrials.gov NCT02866669. Registered on 15 August 2016.

NHLBI AWARD number: PCS-1UH3HL130691.

Contributions to the literature

- PFs documented time spent implementing QI activities in rural primary care practices, providing insights to implementation science researchers on how to operationalize efficient practice facilitation services.
- These data illustrate how PFs organized the time spent to guide practices to implement key driver activities within a 12-month period.
- This paper adds clarity to how PFs allocate their time and resources to support QI efforts. The lack of correlations with common practice characteristics supports engaging in future work to understand other variables that may impact the time facilitators spend to guide practices in change activities, such as staffing needs, supply–demand, and co-location of facilitators and the primary care practices they serve.

## Background

Practice facilitation is a promising intervention to implement practice-level quality improvement (QI) and research projects and has been associated with improved outcomes for patients with a variety of health conditions [1–4]. Practice facilitation is rooted in the principles of evidence-based practice and is purposely dynamic to allow implementation scientists, practice facilitators, and practice staff to tailor services to practices’ specific needs [2, 5, 6]. Practice facilitation methods may vary depending on the scope of the project, practice size, patient population, geographic location, and availiability of funding and resources [7]. The likelihood of an organization’s decision to offer and sustain such services depends on the value the services provide, time required to engage with facilitation services, and associated costs to run such programs. Multiple diverse examples of prior practice facilitation efforts exist, including the Practice Enhancement Assistants (PEAs) employed by the Oklahoma Physicians Resource/Research Network whom facilitate standalone projects at individual practices and contribute to network-wide projects [4]. Other implementation scientists have harnessed practice facilitation’s versatility to enhance colorectal cancer screening in federally qualified health centers (FQHCs) [8]. Similarly, Aledade, Inc. provides support nationwide to primary care practices to help them succeed with value-based care efforts [9]. Quantifying the amount of time practice facilitators (PFs) spend in support of the practices they serve is an important step in developing and implementing strategic planning processes for practice facilitation organizations. Additionally, understanding the variability in time invested by PFs in different tasks when supporting diverse practices is key to setting up business models to test, adapt, and refine practice facilitation over time.

Practice facilitation services support practices in efforts to enhance the quality and efficiency of their clinical services [10, 11]. However, little is known about whether facilitation services are sufficiently resourced to support change activities. We leveraged the work of a research grant to add knowledge of what it takes to provide practice facilitation services to rural primary care practices and to inform a larger strategy of developing a formal practice support program in Alabama. Similarly, we aimed to inform new research questions and projects by investigating the time investments made by facilitators and individuals working in practices receiving practice facilitation. The Southeastern Collaboration to Improve Blood Pressure Control (SEC) was a randomized controlled trial (RCT) aiming to improve blood pressure control among rural African American adults in Alabama (AL) and North Carolina (NC). PF mentors, employed by the NC Area Health Education Center’s (AHEC) practice support program, provided training and guidance on how to implement practice facilitation services in 32 primary care practices. Trained PFs then guided practice staff and providers, using a key driver framework, to implement QI processes and build practice capacity to support ongoing QI activities long after the end of the research study. The PFs utilized a key driver framework to assist practices in moving from knowledge to active learning and ultimately building internal capacity to engage in continuous improvement in practice.

We previously published on the methods that PFs used to guide practices to implement QI activities [12]. In this current manuscript, we examined the amount of time SEC PFs spent performing such tasks with practice stakeholders, including time PFs spent traveling by car in support of practice-level work, working directly on-site with practices, and communicating with practice stakeholders via phone, email, or other remote means. Our hope is that these data can help researchers and business leaders estimate the amount of time and resources that are necessary to optimize the delivery of facilitation services.

## Methods

### SEC trial

The aim of the SEC trial was to understand if a practice- vs. patient-level intervention, alone or in combination, delivered in predominantly rural primary care settings in AL and NC, improved blood pressure control among more than 1,500 African American adult patients with uncontrolled hypertension. The SEC was a 4-armed RCT in which a total of 69 practices in AL and NC were randomized to receive (1) practice-level facilitation (*n* = 16 practices); (2) peer coaching for 25 African American patients with uncontrolled hypertension (*n* = 19 practices); (3) both interventions together (*n* = 16 practices); or (4) enhanced usual care (*n* = 18 practices). Thus a total of 32 rural primary care practices were randomized to the practice facilitation intervention, whose data comprise the basis for this paper. Practices in all study arms received access to an online educational and self-management tool [13], home blood pressure monitors for 25 recruited participants, and other patient-facing educational materials. The results of our main trial have not yet been published.

### Practice facilitation intervention

PFs were expected to visit each of their practices at least once per month over the 12-month intervention period. Facilitators followed a study-specific implementation resource manual to guide practice teams to implement at least one activity from each of 4 “key driver” domains with the goal of having all 4 activities completed within the 12-month time frame. The key activities aimed to (1) use a practice’s own data to drive change; (2) optimize the use of teams to deliver care; (3) standardize care processes such that best practices were implemented; and (4) engage and support patients in their self-management support activities. Four PFs (2 from each state) guided staff members from the practices to implement at least 4 improvement activities using a Plan, Do, Study, Act (PDSA) approach. Details related to how the practice facilitators were trained and how they guided practice-level activities has been previously published [12]. Table 1 includes examples of PDSA activities from each of the four key driver domains in which PFs engaged with their practices. One NC facilitator had a panel of 9 practices while the other had five. One AL facilitator carried out 11 practices over time, while the other managed seven. NC PFs were generally assigned to practices in geographic regions of eastern and central NC, while the AL PFs covered south-central in the east and west of the state.

**Table 1:** Examples of PDSA activities by key driver domain

Key driver	Example activities that facilitators guided practices to implement
Clinical information systems (CIS)	• Developing a practice-level list and monthly reports of patients with HTN and uncontrolled HTN
Standard care processes (SCP)	• Working with staff to document BP in EHRs such that the data would populate HTN reports • Standardizing staff behaviors to repeat BP measurements for patients with SBP^±^ and DBP^±±^ values ≥ 140 and/or ≥ 90 mm Hg
Optimized team care processes (OTC)	• Weekly huddles to identify patients with uncontrolled BP and those requiring extra support; instituting processes for making follow-up calls to check on patient wellbeing and reporting out-of-office BP measurements back to staff
Patient self-management support (SMS)	• Patient use of BP logs to document out-of-office BP’s and instructing clinical staff on how to address noted BP trends that may be related to stress, lack of medication adherence, etc • Use of “Teach Back” methods and goal setting with patients • Providing a raffle in clinic waiting rooms to receive a home BP monitor as a way to promote home monitoring and self-management

The key drivers of implementation is a framework to move from knowledge to implementation of specific interventions. This table demonstrates the four key driver domains and supporting activities for Plan, Do, Study, Act (PDSA) framework in the practice facilitation intervention

*BP* blood pressure, ^±^*SBP* systolic blood pressure, ^±±^*DBP* diastolic blood pressure, *HTN* hypertension

### This study

**Fig. 1:**
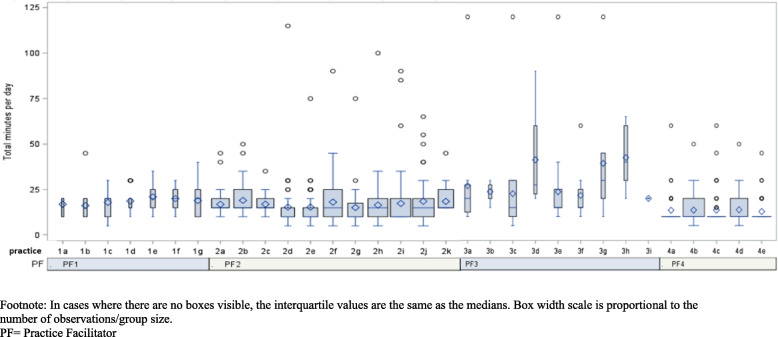
Total minutes of remote communications over 12 months by practice and facilitator. Footnote: In cases where there are no boxes visible, the interquartile values are the same as the medians. Box width scale is proportional to the number of observations/group size. PF, practice facilitator

**Fig. 2:**
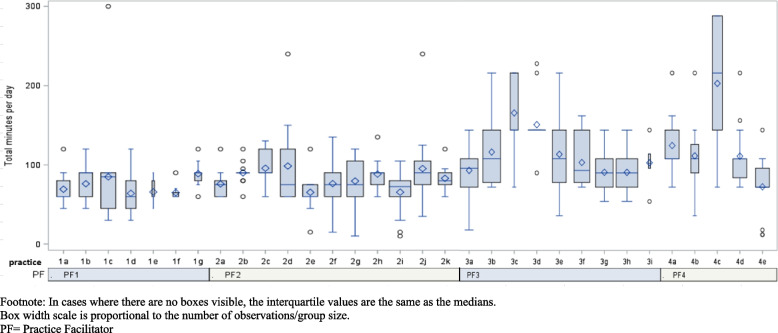
Total minutes on-site over 12 months by practice and facilitator. Footnote: In cases where there are no boxes visible, the interquartile values are the same as the medians. Box width scale is proportional to the number of observations/group size. PF, practice facilitator

We collected practice characteristics as part of the parent trial (Table 2). We asked PFs to document the time they spent (1) driving to and from practices; (2) visiting with practice personnel on-site; and (3) communicating with clinic personnel by phone, email, or video conference (remote communications time) (Figs. 1 and 2 and Table 3). These data were entered as “minutes” per activity into the study database by the PFs after completing tasks. To understand “who” at the practices engaged with PFs, we captured the number of contacts PFs had with providers, staff, and other clinic personnel; time spent with providers (physicians and advanced practice providers) was analyzed separately from time spent with clinical staff members (practice managers, nurses, medical assistants, billing staff, and laboratory personnel). Based upon feedback from our PFs regarding how phone and email communications were often used in tandem and synergistically, and that very little remote video conferencing was used, we aggregated these three items into one time category called “remote” communications. As the trial was ongoing during the early phases of the COVID-19 pandemic, when travel and engagement with practices were limited, we provide these data for all 32 practices but also provide this data limited to the 28 practices that had completed their 12-month practice facilitation intervention before March 2020 (Table 4). We utilized the Template for Intervention Description and Replication (TIDieR) checklist and guide to enhance the quality of intervention reporting for this study.

**Table 2:** Practice characteristics

Practice characteristics	*N*/mean (%, or range)^a^
Full-time providers MD/DO, NP, PA)	3.7 (0–21)
Part-time providers (MD, DO, NP, PA)	0.9 (0–18)
Other (nurses, administrative/clinical support staff)	12.3 (2–53)
Practice ownership type	
Private	11 (34.4%)
FQHC or similar	15 (46.9%)
Free Clinic	1 (3.1%)
Part of a hospital/health system	5 (15.6%)
Years of practice has been in operation	16 (1–42)
Payer Mix	
Medicare	22% (0–60%)
Medicaid	23% (0–50%)
Dual Medicare/Medicaid	8.5% (0–30%)
HMO, PPO, Commercial	21% (0–72%)
Uninsured	24% (0–100%)
Other	2% (0–17%)
Race/ethnicity	
% African American patients	57.5% (25–94%)
% Hispanic Latino patients	10% (0–60%)

^a^Data provided as absolute numbers or means for continuous variables and proportions [14]

**Table 3:** Time spent by facilitators across time categories — all 32 practices randomized to practice facilitation

	Type of contact or activity	Number of observations	Mean # of minutes per observation (SD^c^)	Median # of minutes per observation	Range of minutes per observation	Mean time spent per practice in minutes **(hours)**	Range of minutes **(hours)**
**PF1 UAB 1** **(7 practices)**	**Drive time**	75	105 (23)	105	20–150	1123.6 (18.7)	675–1725 (11.3–28.8)
	**On-site**	74	74 (34)	65	30–300	785.7 (13.1)	395–1190 (6.6–19.8)
	**Phone/email**	133	19 (7)	20	5–45	354.3 (5.9)	220–440 (3.7–7.3)
	**Remote access**	0	0	0	0	0	0
**PF2 UAB 2** **(11 practices)**	**Drive time**	156	149 (57)	150	20–240	2110 (35.2)	960–3510 (16–58.5)
	**On-site**	155	83 (31)	75	10–240	1163.6 (19.4)	910–1430 (15.2–23.8)
	**Phone/email**	639	17 (11)	15	5–115	995 (16.6)	705–1295 (11.8–21.6)
	**Remote access**	2	25 (0)	25	25–25	25 (0.4)	25–25 (0.4–0.4)
**PF3 ECU** **(9 practices)**	**Drive time**	126	117 (83)	70	40–300	1643.9 (27.4)	315–3600 (5.3–60)
	**On-site**	121	94 (34)	90	15–190	1260 (21.0)	600–1885 (10–31.4)
	**Phone/email**	79	27 (26)	20	5–120	240.6 (4)	20–450 (0.3–7.5)
	**Remote access**	3	75 (40)	60	45–120	112.5 (1.9)	45–180 (0.8–3)
**PF4 UNC** **(5 practices)**	**Drive time**	59	137 (65)	120	60–270	1614 (26.9)	610–2640 (10.2–44)
	**On-site**	60	102 (53)	90	10–240	1226 (20.4)	785–1860 (13.1–31)
	**Phone/email**	275	13 (8)	10	5–60	740.4 (12.3)	690–857 (11.5–14.3)
	**Remote access**	0	0	0	0	0	0

**Table 4:** Time spent by facilitators — 28 practices that completed the PF intervention by March 2020

`	Type of contact or activity	Number of observations	Mean # of minutes per observation (SD^c^)	Median # of minutes per observation	Range of minutes per observation	Mean time spent per practice in minutes **(hours)**	Range of minutes **(hours)**
**PF1 UAB 1** **(4 practices)**	**Drive time**	52	111 (20)	120	70–150	1448.8 (24.1)	990–1725 (16.5–28.8)
	**On-site**	51	74 (40)	60	30–300	946.3 (15.8)	705–1190 (11.8–19.8)
	**Phone/email**	69	17 (7)	20	5–45	301.3 (5)	220–430 (3.7–7.2)
	**Remote access**	0	0	0	0	0	0
**PF2 UAB 2** **(11 practices)**	**Drive time**	156	149 (57)	150	20–240	2110 (35.2)	960–3510 (16–58.5)
	**On-site**	155	83 (31)	75	10–240	1163.6 (19.4)	910–1430 (15.2–23.8)
	**Phone/email**	639	17 (11)	15	5–115	995 (16.6)	705–1295 (11.8–21.6)
	**Remote access**	2	25 (0)	25	25–25	25 (0.4)	25–25 (0.4–0.4)
**PF3 ECU** **(8 practices)**	**Drive time**	119	117 (83)	70	40–300	1810 (30.2)	720–3600 (12–60)
	**On-site**	114	94 (34)	90	15–190	1342.5 (22.4)	1135–1885 (18.9–31.4)
	**Phone/email**	78	27 (26)	20	5–120	268.1 (4.5)	95–450 (1.6–7.5)
	**Remote access**	3	75 (40)	60	45–120	112.5 (1.9)	45–180 (0.8–3)
**PF4 UNC** **(5 practices)**	**Drive time**	59	137 (65)	120	60–270	1614 (26.9)	610–2640 (10.2–44)
	**On-site**	60	102 (53)	90	10–240	1226 (20.4)	785–1860 (13.1–31)
	**Phone/email**	275	13 (8)	10	5–60	740.4 (12.3)	690–857 (11.5–14.3)
	**Remote access**	0	0	0	0	0	0

### Statistical analysis

We used descriptive statistics to characterize the amount of time, in minutes and/or hours, spent by different PFs within their practices using the 3 different categories (travel, on-site activities, and remote communications). We used boxplots to visualize the distributions/shape and variability of the data. We also calculated the total amount of time spent in 12 months for each of the three time categories.

Additionally, we conducted paired *t* tests and calculated Pearson r correlation to explore possible correlations between time spent on PF activities (excluding travel time) and practice characteristics such as payer mix, numbers of staff that support individual clinicians, and if the practice was a federally qualified health center (FQHC) or not. To address provider support, we created a provider to staff support ratio measure. For payer correlations, we created a variable to capture the percentage of a practice’s patients with Medicaid and those who were uninsured. The staff-provider ratio variable was dichotomized using median split of less than or equal to 3, and the percent of uninsured or with Medicaid variable was dichotomized using median split of less than or equal to 40 percent. Statistical significance was determined using a two-sided *p*-value < 0.05 for associations between practice characteristics and PF time spent (sans travel time).

## Results

Fifteen of the 32 practices that received practice facilitation services were community health centers, and practices averaged 3.7 fulltime providers. Nearly 25% of patients across all 32 practices were Medicaid beneficiaries, and 58% of the practices’ patients identified as African American (Table 2). Time spent for the three activity categories is presented in Table 3 (all 32 practices) and Table 4 (28 practices not impacted by the COVID-19 pandemic). “Remote time” and “on-site time” are visualized as boxplots in Figs. 1 and 2.

PFs, in aggregate, made 416 trips to practices throughout the study and had 410 face-to-face visits with clinic personnel. The most common activity between PFs and clinic personnel was communicating via remote means (*n* = 1131). The number of encounters and average time spent on activities were similar when the four practices impacted by COVID-19 were excluded (Table 4). PFs spent an average of just over 130 min (~ 2.2 h) driving to practices in these rural regions for individual site visits. The average number of minutes spent per on-site visit was 87 (SD 37) min (range of 10 to 300 min) (Table 3). Over the course of the 12-month intervention, the mean number of hours spent on-site was 18.6 (SD 5.5) with a range from 6.6 to 31.4 h. An average of 17 (SD 12) min was spent each time PFs communicated remotely with practices.

**Fig. 3:**
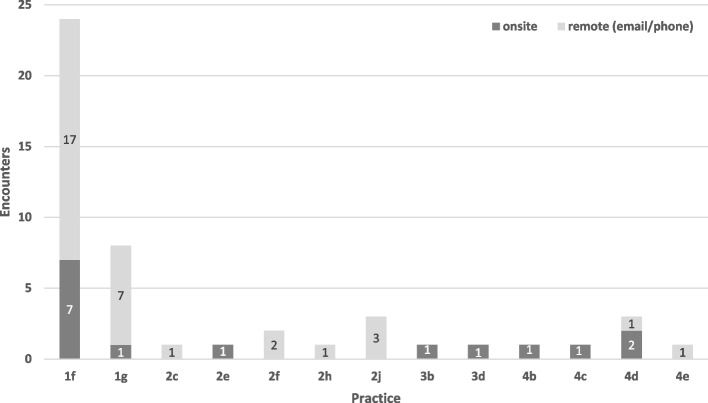
Encounters between PCPs and PFs (on-site or remote communications) in the SEC study (*n* = 13 practices). Footnote: PCPs, primary care providers; PFs, practice facilitators; SEC, Southeastern Collaboration to Improve Blood Pressure Control

Regarding “who” PFs spent time with at practices, the majority of their time was dedicated to working with clinical staff (*n* = 886 encounters) or practice managers alone (*n* = 670 encounters). There were only 48 observations where the facilitator spent time solely with primary care providers (Fig. 3). One-on-one provider-facilitator communications occurred in only 13 of the 32 practices and most of those communications (33 of 48) occurred via remote means.

We did not find any statistically significant differences between the average number of minutes spent by PFs at practices with a staff-provider ratio less than 3 compared to those with a ratio higher than 3 (1703.1 min vs. 1779.3 min; *p*-value = 0.7), practices with a percent of uninsured or Medicaid patients less than 40 vs. > 40% (1700.8 vs. 1757.8 min; *p*-value = 0.8), or whether a practice was an FQHC vs. not (1748.8 min vs. 1709.8 min; *p*-value = 0.8). The Pearson correlation coefficients for the associations between time spent with facilitators and practices’ percent of Medicaid or uninsured patients and staff-provider ratios were *r* = 0.02 and *r* =  − 0.05, respectively.

## Discussion

We leveraged the SEC trial to understand better the time PFs spent with primary care practices to help decision-makers consider how to support a practice facilitation program in Alabama using an NC-based program and mentorship as a model. More time was spent on driving than any other activity, indicating how the proximity of PFs to practices is important when determining efficiencies. Our results suggest it could be strategic to hire facilitators who live as close as possible to the practices they serve, but we recognize that this may not routinely be feasible. Similarly, PF service catchment areas often span large geographical regions, an issue which may be even more challenging when serving practices in rural locations. Conservative estimates based on our data suggest that interventions may need to include time travel of up to 300 min (5 h) per round trip for a single practice visit and up to 60 h per year for travel to support a single rural practice. Efficiencies may be enhanced if more than one practice can be visited in a single trip and or if a hybrid of on-site and remote communications can be organized in the same day. Real-time data collection, using technologies like voice activation software, could optimize efficient data capture and data quality.

Although specific facilitation tasks and time required to complete such tasks may vary by project, we believe these data can inform decision-making as to how practice facilitation services are designed and budgeted even outside of a high blood pressure focus. We posit that it is wise to consider the high-end of time ranges when planning and budgeting PF interventions due to the many competing demands of facilitators and practices. For instance, one PF shared how she spent a relatively excessive amount of time on emails with one practice due to frequent leadership and staff turnover in the practice. She noted that email was the only reasonable option to store such information so that the newer practice stakeholders could have it available while people were transitioning on and off the QI team.

Time spent on-site was the second highest time category, which aligns with the study’s aim of having PFs interact with practice staff at least monthly on-site. Notably, the PFs shared throughout the study that time spent on-site, especially in the early phases of the intervention’s implementation, was critical to successful relationship building and subsequent implementation of change activities. This observation has financial implications for practice facilitation programs, which may result in higher operating expenses in rural areas.

When not traveling to practices, PFs spent significant time communicating with practice staff to support the implementation of QI activities via phone, email, or video conference. Although anecdotally we have learned how skills with using remote communications were enhanced when practices needed to provide more remote care during the COVID-19 pandemic, how much time practices may want to participate in virtual facilitation going forward is unknown. One of our PFs noted that practices were more likely to cancel PF visits when they were scheduled as virtual vs. when the facilitator was known to be driving to engage with practice staff. In this regard, in-person visits to practices remain extremely valuable in maintaining practice relationships and ensuring the successful implementation of the intervention. It may be possible to frontload in-person visits to practices and rely on remote communications after a relationship is established, but this requires further study.

Our results did not find any notable correlations between the average number of minutes PFs provided to a practice (excluding travel time) and common practice characteristics. These results may stimulate researchers and PF organizations to design studies to identify how other variables may be associated with time investments.

## Limitations

These data are limited in that time spent was self-reported by PFs on a monthly basis and often estimated retrospectively. Similarly, PFs were not required to document the time they individually devoted to implementing specific PDSA activities, but rather they tracked their time driving to practices, working on-site, communicating via phone or email, and working remotely. Time tracking software such as *Harvest* or *Timesheet* could be useful in future projects to improve the precision of time estimates and to allow for better data capture of different types of tasks PFs perform. A second limitation is that we did not collect the amount of time that practice staff worked on change activities in between contacts with their facilitators. Our work was focused mainly on activities to address hypertension, but based upon feedback from our facilitators who have worked with practices in improving outcomes for other conditions, we believe that these time estimates may apply reasonably to other conditions.

Finally, the onset of the COVID-19 pandemic during the later years of the trial presented unanticipated challenges. However, during the course of this trial, there were other significant events (hurricanes, ice storms, floods) that also impacted the cadence of facilitation work with practices. These realities only further support using ranges of data when anticipating budget and other resource needs to support facilitation interventions.

## Conclusion

These time estimates captured by PFs working with 32 primary care practices that serve rural patients in the SEC trial may uniquely offer stakeholders guidance regarding PF trial design and program budgeting. Our data demonstrate that practice facilitation, as configured in the SEC trial, is a time-intensive activity, and co-location of facilitators and the primary care practices they serve may be valuable. Similarly, attempts to increase the effectiveness of remote communications and reduce the time dedicated to driving should be considered in order to enable PFs to help practices implement change activities in a constantly changing environment. By sharing this work with other researchers and stakeholders interested in practice facilitation and QI, we hope to provide clarity regarding the resources needed to support facilitation services and to inspire others to investigate ways to optimize practice facilitation services and outcomes.

## Data Availability

The datasets used and/or analyzed during the current study are available from the corresponding author on reasonable request.

## Abbreviations

PF: Practice facilitator
PFs: Practice facilitators
QI: Quality improvement
RCT: Randomized controlled trial
AL: Alabama
NC: North Carolina
PDSA: Plan, Do, Study, Act
SEC: Southeastern Collaboration to Improve Blood Pressure Control
FQHC: Federally qualified health center
